# Potentiometric and Blood Plasma Simulation Studies of Nickel(II) Complexes of Poly(amino)amido Pentadentate Ligands: Computer Aided Metal-Based Drug Design

**DOI:** 10.1155/2014/863612

**Published:** 2014-10-12

**Authors:** Sebusi Odisitse, Graham E. Jackson

**Affiliations:** ^1^Department of Chemistry, University of Cape Town, Private Bag X3, Rondebosch, Cape Town 7701, South Africa; ^2^Natural Resources and Materials, Botswana Institute for Technology Research and Innovation, Plot 50654 Machel Drive, Private Bag 0082, Gaborone, Botswana

## Abstract

The thermodynamic equilibria of nickel(II) with N,N′-di(aminoethylene)-2,6-pyridinedicarbonylamine (L1), Bis-(N,N-dimethylethyl)-2,6-pyridinedicarboxamide (L2), and N,N′-bis[2(2-pyridyl)-methyl]pyridine-2,6-dicarboxamide (L3) have been studied at 25°C and an ionic strength of 0.15 mol dm^−3^ by glass electrode potentiometry. The protonation and formation constants added to blood plasma model predict that Cu(II) competes effectively against Ni(II), Zn(II), and Ca(II) for these ligands *in vivo*.

## 1. Introduction

Metal ions may be part of the active sites of enzymes, stabilise the macromolecular structure of proteins, and affect enzymes or membranes to control cell metabolism [1]. Nickel has long been recognised as an essential constituent of the catalytic center of four different types of enzymes, urease in plants, hydrogenase, CO dehydrogenase, and methyl CoM reductase in some strains of bacteria [2]. For many years, there has been great interest in the study of complexes able to mimic active sites of metalloproteins.

**Figure 1:**
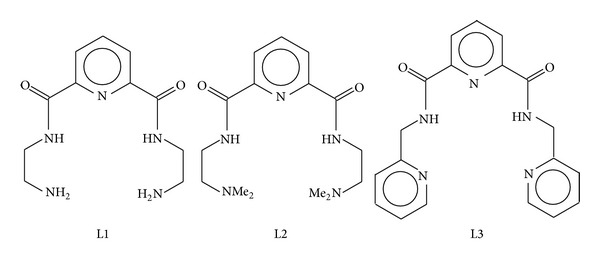
Schematic structures of ligands (L) studied.

We have recently investigated the solution chemistry of Cu^2+^, Zn^2+^, and Ca^2+^ with N,N′-di(aminoethylene)-2,6-pyridinedicarbonylamine (L1), Bis-(N,N-dimethylethyl)-2,6-pyridinedicarboxamide (L2), and N,N′-bis[2(2-pyridyl)-methyl]pyridine-2,6-dicarboxamide (L3) (Figure 1) for possible use as copper-based anti-inflammatory drugs in the treatment of rheumatoid arthritis [3, 4]. Zn^2+^ and Ca^2+^ were included in the study as they are present in high concentration* in vivo* and so are potential competitors of Cu^2+^. The potentiometric results indicated that copper(II) formed stable complexes with all these ligands. Blood plasma simulation studies showed that L3 is better at mobilising Cu^2+^ than L1 and L2. Biodistribution experiments on mice revealed that the copper(II) complexes of these ligands have much longer biological half-lives than those studied by Jackson et al. [5].

Although the concentration of free nickel(II) in blood plasma is negligible [2], a high intake of nickel could affect the concentration of the administration of copper(II) antiarthritic agents. With this in mind, we have investigated nickel(II) complexes of all these three ligand systems in solution. Potentiometry was used to calculate formation constants of these ligands with Ni^2+^ and blood plasma modelling was used to predict the ability of the metal to alter the bioavailability of copper complexes previously investigated [3, 4].

## 2. Experimental

The ligands L1, L2, and L3 were synthesised as described previously [3, 4]. They were characterised spectroscopically and by elemental analysis.

All solutions for potentiometry were prepared in glass distilled water which had been boiled and cooled under an inert atmosphere to remove dissolved carbon dioxide. Recrystallized NaCl was used as background electrolyte at an ionic strength of 0.15 mol dm^−3^ (Cl^−^). Nickel(II) chloride, HCl, NaOH, and EDTA (Merck) were commercially available and of analytical grade. These were used without purification. The 0.1 mol dm^−3^ solutions of NaOH and HCl were prepared from Merck Titrisol ampoules and standardized by titrating with potassium hydrogen phthalate (KHP) and sodium tetraborate decahydrate (Borax), respectively. The NaOH solutions were further standardized against the standard HCl solution. Acid-base titration was also used to check carbonate contamination of the sodium hydroxide titrant solution using the Gran method [6]. The standardised solutions were used within a short period of time after preparation and were discarded whenever there were signs of carbonate contamination.

The potentiometric titrations were performed under an inert atmosphere of purified nitrogen, at 25°C, using Radiometer PHM 84 research pH meter and a Metrohm Dosimat 665 piston burette, which were controlled by a computer. The pH meter was equipped with a Metrohm 6.0222.100 glass electrode, its slope was determined from a buffer line and the response intercept, *E*°, was determined* in situ*. Titrations were performed in the pH range 2–11 in the concentration range 3 × 10^−4^–1 × 10^−3^ mol dm^−3^ at metal ion to ligand ratios ranging between 1 : 1 and 1 : 3. The data were analysed using the ESTA suite of computer programs [7]. Various speciation models were tried based on the experimental *Z*_*M*_-bar and *Q*_*M*_-bar curves. The water ionization constant (p*K*_*w*_) used was 13.73. The protonation constants of the ligands and the overall formation constants of the Ni(II) systems were computed using general expressions given as follows:

(1) $$\begin{array}{c} p\text{M}+q\text{L}+r\text{H}⟶{\text{M}}_{p}{\text{L}}_{q}{\text{H}}_{r} \end{array}$$

(2) $$\begin{array}{c} \beta_{pqr}=\frac{\left[{\text{M}}_{p}{\text{L}}_{q}{\text{H}}_{r}\right]}{{\left[\text{M}\right]}^{p}{\left[\text{L}\right]}^{q}{\left[\text{H}\right]}^{r}}, \end{array}$$

where *p*, *q*, and *r* are stoichiometric coefficients of the components in the complex.

Blood plasma modelling was performed using a blood plasma equilibrium database consisting of 10 metal ions and more than 5000 ligands [8]. To this database, the protonation and Ni(II) formation constants determined in this study were added. The database was used together with typical constituent concentrations of blood plasma [8] to calculate the speciation of the system and the plasma mobilizing index (pmi) of the ligand. This was achieved using the ECCLES (Evaluation of Constituent Concentration in Large Equilibrium Systems) program [9]. Pmi is a measure of the ability of a ligand to move metal ion from a protein bound form to a low molecular weight form. It is defined by

(3) pmi=(total  concentration  of  low-molecular-weightmetal  complex  species  in  the  presence  of  drug) ×(total  concentration  of  low-molecular-weightmetal  complex  species  in  normal  plasma)−1.

## 3. Results and Discussion

### 3.1. Potentiometry

The three measurable acid dissociation (protonation) constants corresponding to the pyridyl nitrogens and terminal amino groups of L1, L2, and L3 are given in Table 1. The first protonation constant (p*K*_*a*1_ = log⁡*β*_011_) corresponds to the protonation of the terminal amine or pyridyl nitrogen. The p*K*_a_ values of pyridine and 2-methylpyridine are reported as 5.34 and 6.14, respectively, [10] whereas the introduction of a carbonyl group in 2-methylpyridine resulted in a p*K*_*a*_ value of 2.66 [11]. Thus, a p*K*_*a*1_ of 4.62 is reasonable for L3. Comparing L1 and L2, one may expect the addition of two methyl groups in L2 to increase p*K*_*a*1_ through the methyl inductive effect. However, in aqueous solution, the entropy of hydration outweighs the inductive effect leading to the observed p*K*_*a*1_ of L1 > p*K*_*a*1_ of L2 [12]. Since each ligand is symmetrical, one could expect the second protonation constant p*K*_*a*2_(log⁡*β*_012_ − log⁡*β*_011_) to be the same as the first protonation constant, p*K*_*a*1_. However, the second proton is added to a ligand that is already protonated and so electronic repulsion makes addition of the second proton more difficult. In addition, there is an entropic factor; for the first protonation step, there are two equivalent sites available but, for the second protonation, there is only one site, the other already being occupied. Jackson et al. reported p*K*_*a*1_ and p*K*_*a*2_ values for N,N′-bis[aminoethyl] ethanediamne, that is, H_2_N-CH_2_-CH_2_-NH-C(O)-C(O)-NH-CH_2_-CH_2_-NH_2_, (p*K*_*a*1_ = 9.31 and p*K*_*a*2_ = 8.43) and N,N′-bis[2-(dimethylamino)-ethyl] ethanediamide, that is, Me_2_N-CH_2_-CH_2_-NH-C(O)-C(O)-NH-CH_2_-CH_2_-NMe_2_, (p*K*_*a*1_ = 8.72 and p*K*_*a*2_ = 7.92) [12]. The low value of the third protonation constant (p*K*_*a*3_ = log⁡*β*_013_ − log⁡*β*_012_) of 2.03, 1.74, and 1.98 corresponding to the central pyridyl nitrogen for L1, L2, and L3, respectively, is due to the electronic repulsion effect and the base weakening effect of the adjacent amide groups [13]. Therefore, it is expected that the protonation constant corresponding to the central pyridyl nitrogen in L1, L2, and L3 should decrease further when two amide groups are present.

**Figure 2:**
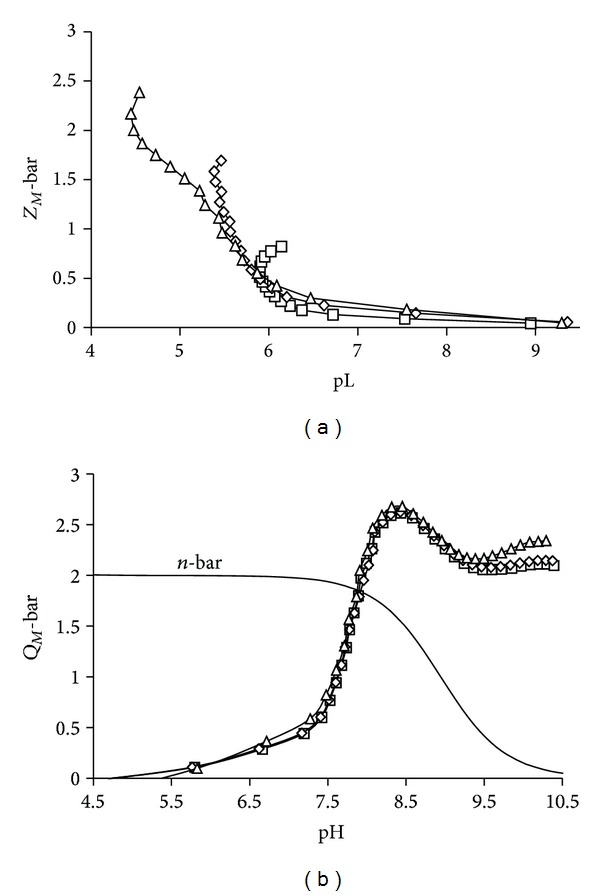
Experimental and theoretical (solid line) (a) formation function and (b) deprotonation function curves for Ni(II)-L1 system at 25°C and an ionic strength 0.15 M (Cl^−1^). M : L ratios 1 : 1 (□), 1 : 2 (◊), and 1 : 3 (Δ) are displayed. The theoretical line was calculated using model 1 given in Table 1.

The complex formation (*Z*_*M*_-bar) and the deprotonation (*Q*_*M*_-bar) functions [3, 4, 14, 15] derived from the experimental data have been used to visualize the experimental data and to decide on the speciation model. The *Z*_*M*_-bar function measures the number of ligands bound per metal ion while *Q*_*M*_-bar indicates the number of protons released upon metal ion complexation. The two functions are derived from the free and total concentrations of the participating components as well as the protonation constants of the ligands. The classical complex formation function should level at a *Z*_*M*_-bar value of 1 for mononuclear ML species formation. However, deviations from ideal behaviour are indicative of the different speciation occurring in solution.  Figure 2(a) shows the *Z*_*M*_-bar function plotted against pL (−log⁡[L]) for Ni(II)-L1. The *Z*_*M*_-bars do not level off at 1 indicating that ML is not the only species in solution. The splitting of the curves for different metal to ligand ratios is due to the presence of polynuclear or protonated species, while the fanning back of the curves is indicative of hydroxo- and/or mixed hydroxospecies formation. The same pattern was observed for all the three systems and is typical of amide coordination.

The *Q*_*M*_-bar function (Figure 2(b)) rises at pH 4.5, the start of complexation, and rises from 0 to about 2.7 (*n*-bar > 2) indicating that, approximately, three protons have been released due to complexation. The *n*-bar function measures the average number of protons that would be bound to the ligand in the absence of the metal ion. The release of a fourth proton is observed at pH > 9 and this was also found for the L2 system. Early metal assisted deprotonation of the amide groups was observed for the L3 system at pH 6.2, thus forming CuLH_−1_ and/or CuLH_−2_.

The potentiometric data analysis using ESTA suite of programs [7] gave two models for the Ni(II)-L1 and Ni(II)-L2 systems consisting of MLH_−2_, MLH_−1_, and either ML or MLH as shown in Table 1. This posed difficulty in deciding on the model which best describes the system. Both models had low standard deviation and Hamiltonian *R*-factors and described the experimental data equally well as far as statistical criteria were concerned and also on chemical grounds. However, the model with MLH is favoured because of the splitting of the *Z*_*M*_-bar curves at low pH values, Figure 2(a). Without the MLH species, the curves should be superimposable at low pH. For L2, the high standard deviation (0.38) for *β*_110_ also supports selection of model 1. The reproducibility of repeat titrations, low standard deviations, and the Hamilton *R*-factors as well as an excellent agreement between the theoretical and experimental complex formation and deprotonation functions lends confidence to the results for these systems.

**Figure 3:**
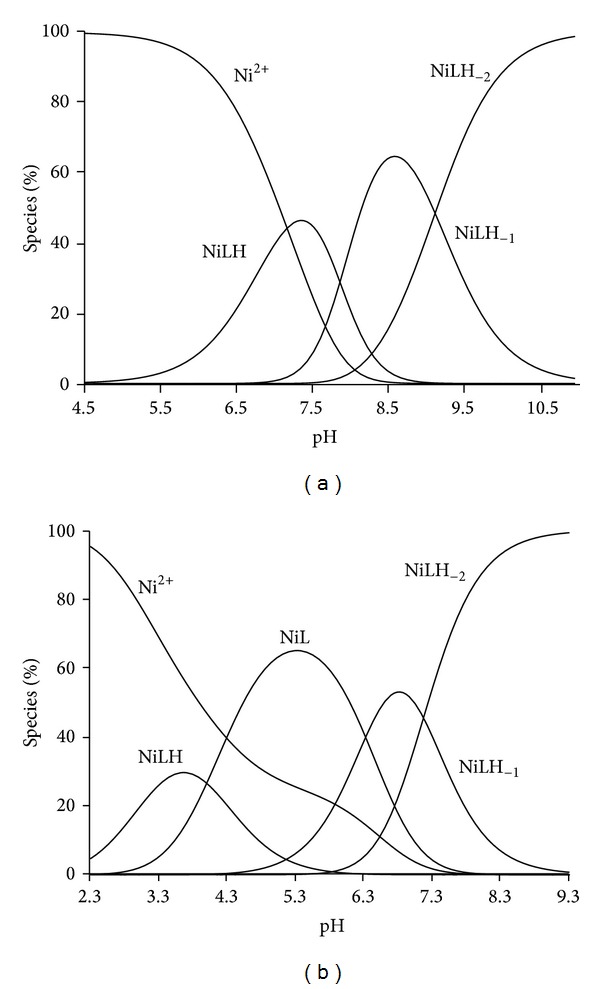
(a) Calculated speciation distribution graphs for a Ni(II)-L1 solution ([M] = 0.00051 mol dm^−3^ and [L] = 0.00101 mol dm^−3^) as a function of pH. (b) Calculated speciation distribution graphs for a Ni(II)-L3 solution ([M] = 0.00051 mol dm^−3^ and [L] = 0.00101 mol dm^−3^) as a function of pH.

The formation constants (log *β*_*pqr*_'s) for the MLH, ML, MLH_−1_, and MLH_−2_ complexes of the Ni(II)-L systems are given in Table 1. These values are lower than the corresponding values for the Cu (II)-L1, Cu(II)-L2, and Cu(II)-L3 systems [3, 4]. Zn(II) and Ca(II) were also found to form reasonably stable complexes with these systems but their stabilities are lower than that of Ni(II). The order of stability Cu(II) > Ni(II) > Zn(II) > Ca(II) is as expected [16]. Although Ni(II) is a weaker acid than Cu(II), high intake of this essential metal ion could potentially disrupt or alter the bioavailability of copper complexes administered as antiarthritic agents. The speciation distribution curves given in Figure 3(a) show a mixture of species at a physiological pH of 7.4 for L1 and this was also observed for L2 whereas L3 showed MLH_−2_ as the predominant species at this pH as shown in Figure 3(b). The reason for this, we believe, is the preformed structure of L3 which facilitates metal assisted deprotonation of the amide.

**Figure 4:**
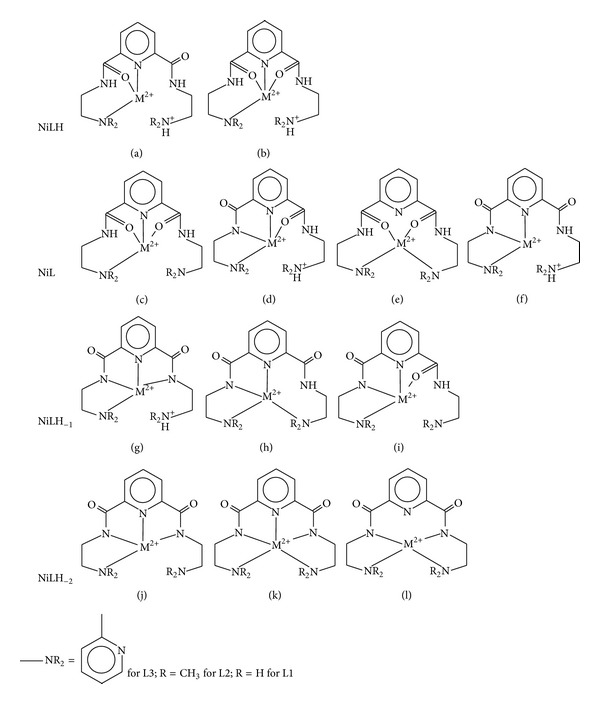
Schematic representation of proposed structures of the various Nickel-ligand species.

From potentiometric data given in Table 1, we have proposed possible structures for the different species in solution, Figure 4. The ligands in this study are based on the pyridine-2,6-dicarboxamide moiety to which have been added two ethylene linkages. Once deprotonated, the pyridine-2,6-dicarboxamide unit should be planar and maintain planarity in various metal complexes [17–19]. The basal plane is defined by the N_amido_-N_py_-N_amido_ coordination of the ligand. The central pyridine nitrogen acts as an anchor for amide coordination and it is also the first one to be coordinated to the metal, thus allowing the metal to be in close proximity for the amide ionization. Two possibilities for the MLH species are shown in Figures 4(a) and 4(b). Between the two structures, Figure (a) seems the more likely coordination geometry because Figure (b) would be destabilised by the rigidity of the pyridine moiety when both carbonyl oxygens are coordinated. It is expected that as the pH increases, there would be a transition from Ni–O to Ni–N coordination as Ni(II) would induce ionization of the amide protons. From the four proposed structures of ML, Figures 4(c)–4(f), Figure 4(f) is favoured because of the two 5-membered chelate rings that are formed. As for MLH_−1_, structure Figure 4(g) is preferred over Figures 4(h) and 4(i). Brooker et al. have investigated 2,6-bis(1-propanecarboxamido-3-amino)pyridine (a five nitrogen donor ligand) which is analogous to ligands in our study [20, 21]. In the solid state, Ni(II) had a square planar geometry with four coordinated nitrogen donor atoms. The fifth nitrogen from the terminal amino groups was uncoordinated and protonated. We propose a similar geometry for the MLH_−2_ species of L1-3 with one uncoordinated terminal amino/pyridine group, Figure 4(j). In the solid state, Ni(II)-L3 system has been reported to form a trinuclear complex as the major species and a monomeric complex with four nitrogen donor coordination arrangements and uncoordinated terminal pyridine group as the minor species [17, 22].

### 3.2. Blood Plasma Simulation

In order to determine the* in vivo* mobilization of Ni(II) by L1, L2, and L3, blood-plasma modelling [8, 9] was used to estimate the ability of these ligands to mobilize Ni(II). This was done by calculating the plasma mobilizing index (pmi) of the ligand using a thermodynamic model of blood plasma. The pmi of a metal ion by a ligand is defined as the fractional increase in the total concentration of low molecular mass (lmm) complexes of the metal ion caused by the ligand. This takes into account competition between the ligand and all the endogenous metal ions and low molecular mass ligands present in the blood plasma model. In calculating the pmi curves, use has been made of the ECCLES model [3, 4] of blood plasma to which formation constants determined in potentiometric study have been added.

**Figure 5:**
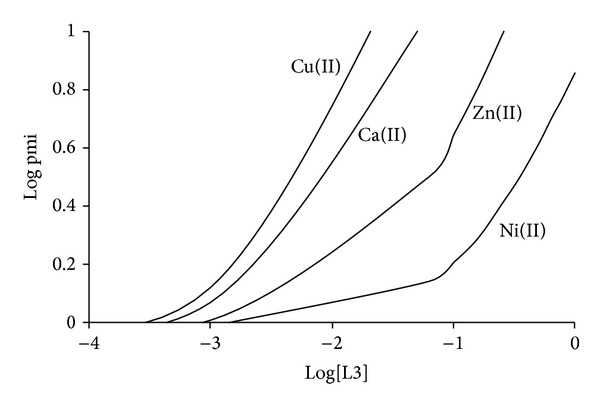
Logarithms of Cu(II), Ni(II), Zn(II), and Ca(II) plasma mobilizing index plotted against log⁡[L3].

Inspection of Figure 5 shows that L3 is very poor at mobilizing Ni(II) in blood plasma and in fact mobilizes significantly more Cu(II), Ca(II), and Zn(II). A similar trend was observed for L1 and L2. The selectivity factors are in the order Cu(II)/Ca(II) > Cu(II)/Zn(II) > Cu(II)/ Ni(II) which is in accordance with the Irving-Williams series [16].

The poor mobilization of Ni(II) by L1-3 is because the ligands preferentially bind to Cu (II), Ca(II), and Zn(II). The preferential binding to Cu(II) is understandable as Cu(II) forms more stable complexes than Ni(II). However, Ni(II) forms more stable complexes than Zn(II) and Ca(II).* In vivo*, the free concentration of Ca(II) and Zn(II) is 10^15^ and 10^9^ times greater than the free concentration of Ni(II) and this higher concentration means that they are able to displace Ni(II) from its complexes. At a total L3 concentration of 3.1 mmol dm^−3^, the pmi for Ni(II) is very low (<0.1) which means that the use of this ligand to mobilize Cu(II) as an anti-inflammatory agent will not affect the speciation of the essential metal ion, Ni(II). However, they will affect the speciation of Ca(II) and Zn(II).

## 4. Conclusion

The ligands L1, L2, and L3 are quite selective towards Cu(II) over Ni(II) due to the ease with which Cu(II) deprotonates the amide nitrogen groups. The rigidity of the pyridyl moiety and pyridyl nitrogen that acts as an anchor enhanced the ionization of the amide protons in the presence of the Ni(II) ion. Possible geometries for the different species in solution were postulated based on a comparison with literature data. The blood plasma simulation suggests that the speciation of Ni(II) will not be adversely affected by the use of these ligands as anti-inflammatory agents. However, the speciation of Zn(II) and Ca(II) will be adversely affected.

## Figures and Tables

**Table 1:** Formation constants (log⁡*β*_*pqr*_) for Ni(II) complexes of L1, L2, and L3, *β*_*pqr*_ = [M_*p*_L_*q*_H_*r*_]/[M]^*p*^[L]^*q*^[H]^*r*^, *I* = 0.15 mol dm^−3^ (NaCl), and *T* = 25°C.

Ligand	Metal	*p*	*q*	*r*	log⁡*β*_*pqr*_ (*σ*_*pqr*_)	*R* ^*H*^	*R* ^*H*^_lim⁡_
L1	H^+^	0	1	1	9.20 (0.01)	0.01	0.01
		0	1	2	17.88 (0.01)		
		0	1	3	19.91 (0.03)		
	Ni(II)	Model 1					
		1	1	1	13.88 (0.01)	0.02	0.01
		1	1	−1	−1.82 (0.02)		
		1	1	−2	−10.92 (0.02)		
		Model 2					
		1	1	0	6.08 (0.04)	0.04	0.02
		1	1	−1	−2.00 (0.04)		
		1	1	−2	−10.94 (0.04)		
L2	H^+^	0	1	1	8.64 (0.01)	0.01	0.01
		0	1	2	16.72 (0.01)		
		0	1	3	18.46 (0.06)		
	Ni(II)	Model 1					
		1	1	1	11.22 (0.07)	0.02	0.01
		1	1	−1	−4.99 (0.02)		
		1	1	−2	−13.94 (0.01)		
		Model 2					
		1	1	0	2.17 (0.38)	0.02	0.01
		1	1	−1	−5.05 (0.02)		
		1	1	−2	−13.99 (0.01)		
L3	H^+^	0	1	1	4.62 (0.01)	0.01	0.01
		0	1	2	8.13 (0.01)		
		0	1	3	10.11 (0.05)		
	Ni(II)	1	1	1	7.70 (0.08)	0.03	0.02
		1	1	0	3.69 (0.04)		
		1	1	−1	−2.65 (0.04)		
		1	1	−2	−9.84 (0.03)		

*σ*_*pqr*_ denotes standard deviation in log⁡*β*_*pqr*_, and *R* ^*H*^ and *R* ^*H*^_lim⁡_ are the Hamiltonian *R*-factor and its limit. The general formula of a complex is M_*p*_L_*q*_H_*r*_.
